# Comparison of assembly platforms for the assembly of the nuclear genome of *Trichoderma harzianum* strain PAR3

**DOI:** 10.1186/s12864-023-09544-6

**Published:** 2023-08-11

**Authors:** Zachary Gorman, Jianchi Chen, Adalberto A. Perez de Leon, Christopher Michael Wallis

**Affiliations:** Crop Diseases, Pests and Genetics Research Unit, USDA-ARS San Joaquin Valley Agricultural Sciences Center, Parlier, CA 93648 USA

**Keywords:** Genome assembly, Nuclear genome, *Trichoderma harzianum*, Biocontrol, Grapevine

## Abstract

**Background:**

*Trichoderma* is a diverse genus of fungi that includes several species that possess biotechnological and agricultural applications, including the biocontrol of pathogenic fungi and nematodes. The mitochondrial genome of a putative strain of *Trichoderma harzianum* called PAR3 was analyzed after isolation from the roots of Scarlet Royal grapevine scion grafted to Freedom rootstock, located in a grapevine vineyard in Parlier, CA, USA. Here, we report the sequencing, comparative assembly, and annotation of the nuclear genome of PAR3 and confirm its identification as a strain of *T*. *harzianum*. We subsequently compared the genes found in *T. harzianum* PAR3 to other known *T. harzianum* strains. Assembly of Illumina and/or Oxford Nanopore reads by the popular long-read assemblers, Flye and Canu, and the hybrid assemblers, SPAdes and MaSuRCA, was performed and the quality of the resulting assemblies were compared to ascertain which assembler generated the highest quality draft genome assembly.

**Results:**

MaSuRCA produced the most complete and high-fidelity assembly yielding a nuclear genome of 40.7 Mb comprised of 112 scaffolds. Subsequent annotation of this assembly produced 12,074 gene models and 210 tRNAs. This included 221 genes that did not have equivalent genes in other *T. harzainum* strains. Phylogenetic analysis of ITS, *rpb2*, and *tef1a* sequences from PAR3 and established *Trichoderma* spp. showed that all three sequences from PAR3 possessed more than 99% identity to those of *Trichoderma harzianum*, confirming that PAR3 is an isolate of *Trichoderma harzianum*. We also found that comparison of gene models between *T. harzianum* PAR3 and other *T. harzianum* strains resulted in the identification of significant differences in gene type and number, with 221 unique genes identified in the PAR3 strain.

**Conclusions:**

This study gives insight into the efficacy of several popular assembly platforms for assembly of fungal nuclear genomes, and found that the hybrid assembler, MaSuRCA, was the most effective program for genome assembly. The annotated draft nuclear genome and the identification of genes not found in other *T. harzainum* strains could be used to investigate the potential applications of *T*. *harzianum* PAR3 for biocontrol of grapevine fungal canker pathogens and as source of anti-microbial compounds.

## Background

*Trichoderma* is a diverse genus of fungi that includes several species that possess biotechnological and agricultural applications, including the biocontrol of fungi and nematodes and the production of enzymes used in biofuel generation [1–3]. Next generation sequencing approaches have helped to unravel the molecular basis of diversity among different *Trichoderma* spp. and has facilitated the development of new biotechnological applications for agriculture [4]. The mitochondrial genome of a *Trichoderma* isolate, putatively identified as *Trichoderma harzianum* strain PAR3, was previously reported [5]. *T*. *harzianum* PAR3 was isolated from the roots of Scarlet Royal grapevine scion grafted to Freedom rootstock in a vineyard in Parlier, CA, USA. Grapevine trunk diseases (GTD) reduce yield and eventually cause grapevine death [6]. These diseases are caused by fungal canker pathogens that infect grapevines through wounds caused by pruning or scion-rootstock grafting in nurseries prior transplantation into a vineyard [7]. Preliminary studies show that *T*. *harzianum* PAR3 may provide resistance of grapevine to several fungal pathogens that cause GTDs [5]. Thus, *T*. *harzianum* PAR3 could be developed to combat some of these fungal pathogens. Assessing the potential of *T*. *harzianum* strain PAR3 as a biocontrol agent requires understanding the underlying genetic factors that may contribute to the protection of grapevines against GTDs. This study addresses that need by generating a high-quality nuclear genome assembly of *T. harzianum* PAR3.

Many different programs are available for genome assembly and use a diverse array of strategies for assembly. Accordingly, different assemblies generated from the same sequencing data can vary depending on the programs used to generate these assemblies. Assemblies based on short-read sequences, such as those generated by Illumina systems, provide high-fidelity resolution of the genome sequence but lack structural resolving power. Conversely, assemblies based on long-read sequences from Oxford Nanopore Technologies (ONT) instruments provide valuable structural information but have a higher nucleotide error rate than short-read assemblies. PacBio sequencing can provide both long and high-fidelity reads [8] but is more costly than Illumina or ONT sequencing [9]. To overcome the limitations of ONT- and Illumina-only assemblies and to utilize their respective strengths both types of reads can be utilized in a single genome assembly, resulting in high-quality assembly. In general, there two methods are routinely employed to use both short- and long- reads in an assembly. The first is to assemble error-prone ONT reads with long-read assemblers, such as Canu [10] or Flye [11], and then use Illumina reads to polish and correct sequence errors in the assembly. Another approach involves using both Illumina and ONT reads for hybrid genome assembly. SPAdes [12] and MaSuRCA [13] are two prominent assemblers capable of performing hybrid genome assembly, though they both rely on different strategies for generating assemblies. In brief, SPAdes utilizes the de Brujin graph to generate sequences based on short reads and then uses long reads to fill in gaps between these sequences [12], whereas MaSuRCA builds “mega-reads” by combining extended short “super-reads” with long reads and assembling these reads [14].

Herein, we report the use of Canu, Flye, SPAdes, MaSuRCA, a combination of Canu and Flye, and a combination of MaSuRCA and Flye, in conjunction with post-assembly Illumina polishing by Pilon [15] to generate several assemblies of *Trichoderma harzianum* PAR3. All assemblers utilized estimated 28x coverage ONT and 518x coverage Illumina sequencing data. The resulting assemblies were compared, and the highest quality assembly was annotated by Maker [16] and InterProScan [17]. Phylogenetic identification of *Trichoderma* spp., particularly those within the *Harzianum* clade, is difficult due to the close genetic similarity of widely used barcoding sequences, such as the internal transcribed spacer (ITS) regions of ribosomal subunits. In fact, several *Trichoderma* isolates previously considered to be *Trichoderma harzianum* were reclassified as distinct, closely relates species of *Trichoderma* within the *Harzianum* clade [18]. In addition to ITS, a variety of other barcoding sequences have been used for identification, including different fragments of the same gene, which has resulted in inconsistent identifications. As such, new stringent criteria have been established for accurate molecular identification of *Trichoderma* spp [19]. Accordingly, we built upon the previous identification of PAR3 [5] by analyzing the ITS, *rpb2*, and *tef1* barcoding sequences of PAR3 and other *Trichoderma* spp., and confirmed PAR3 is a strain of *Trichoderma harzianum*.

Previously, comparison of gene models between two *T. harzianum* strains isolated from Europe and South America, respectively, revealed substantial differences in number of genes with similarity between these strains, with approximately 10–12% of genes that were unique to the two strains [20]. As *T. harzianum* PAR3 was isolated from North America and differs greatly in total gene number compared to other *T. harzianum* strains, we compared PAR3 genes to those of other *T. harzianum* strains. The analysis revealed a sizable difference in the number of equivalent genes between *T. harzianum* PAR3 and other *T. harzianum* strains and identified many genes that were unique to the PAR3 strain.

## Results

Assembly of only ONT-reads was performed with Canu and Flye, and hybrid assemblies were performed by SPAdes and MaSuRCA. Additionally, Flye was also used to perform assembly of Canu-corrected ONT reads (Canu-Flye) and of MaSuRCA-generated “mega-reads” (MaSuRCA-Flye). The least number of contigs, 101, was produced by Canu-Flye, whereas SPAdes yielded the most contigs, 237 (Table 1). Canu generated the second most contigs, 200, followed by Flye, MaSuRCA-Flye, and MaSuRCA, which produced, 133, 124, 128 contigs, respectively. Contigs were additionally scaffolded within MaSuRCA and SPAdes, generating 115 and 223 scaffolds, respectively. Although, SPAdes produced the most contigs/scaffolds, over half of the scaffolds of small size (< 500 bp) and consisted of simple single- or double-nucleotide repeats. These small scaffolds were likely a result of aberrant reads, and no other assembler produced any similar contigs/scaffolds less than 500 bp in length. Thus, we removed these sequences from the SPAdes assembly, which left 90 scaffolds. We next sought to remove any contigs/scaffolds in the assemblies that belonged to the published PAR3 mitochondrial genome [5]. Mito contigs and/or scaffolds were identified by BLAST searches for the mitogenome of PAR3 and removed from the assemblies. One mitochondrial contig/scaffold was found in the Canu, MaSuRCA-Flye assemblies, with 199 contigs and 123 contigs, remaining in these assemblies. Five mitochondrial scaffolds were removed from the SPAdes assembly, leaving 85 scaffolds, and 95 contigs were left in the Canu-Flye assembly after 6 mitochondrial contigs were removed. Three mitochondrial scaffolds were removed from the MaSuRCA assembly, leaving 112 scaffolds comprising this assembly. The Flye assembly possessed more mitochondrial contigs than any of the other assemblies, 19, with 114 contigs remaining after their removal (Table 1). In addition to mitochondrial sequences largely existing in separate contigs/scaffolds, all assemblies had a small portion of mitochondrial sequence, usually a few thousand basepairs, that was interspersed within two otherwise nuclear contigs/scaffolds. The long-read only assemblers, Canu, Flye, and Canu-Flye, produced smaller genome sizes, 39.1 Mb, 40.1 Mb, and 39.2 Mb, respectively, compared to the hybrid assemblers which produced 41.9 Mb (SPAdes), 40.2 Mb (MaSuRCA-Flye), and 40.7 Mb (MaSuRCA) assemblies. Out of these, MaSuRCA produced the genome size closest to the 41 Mb size of *T. harzianum* CBS226.95, the type strain of *T. harzianum* [21].

**Fig. 1:**
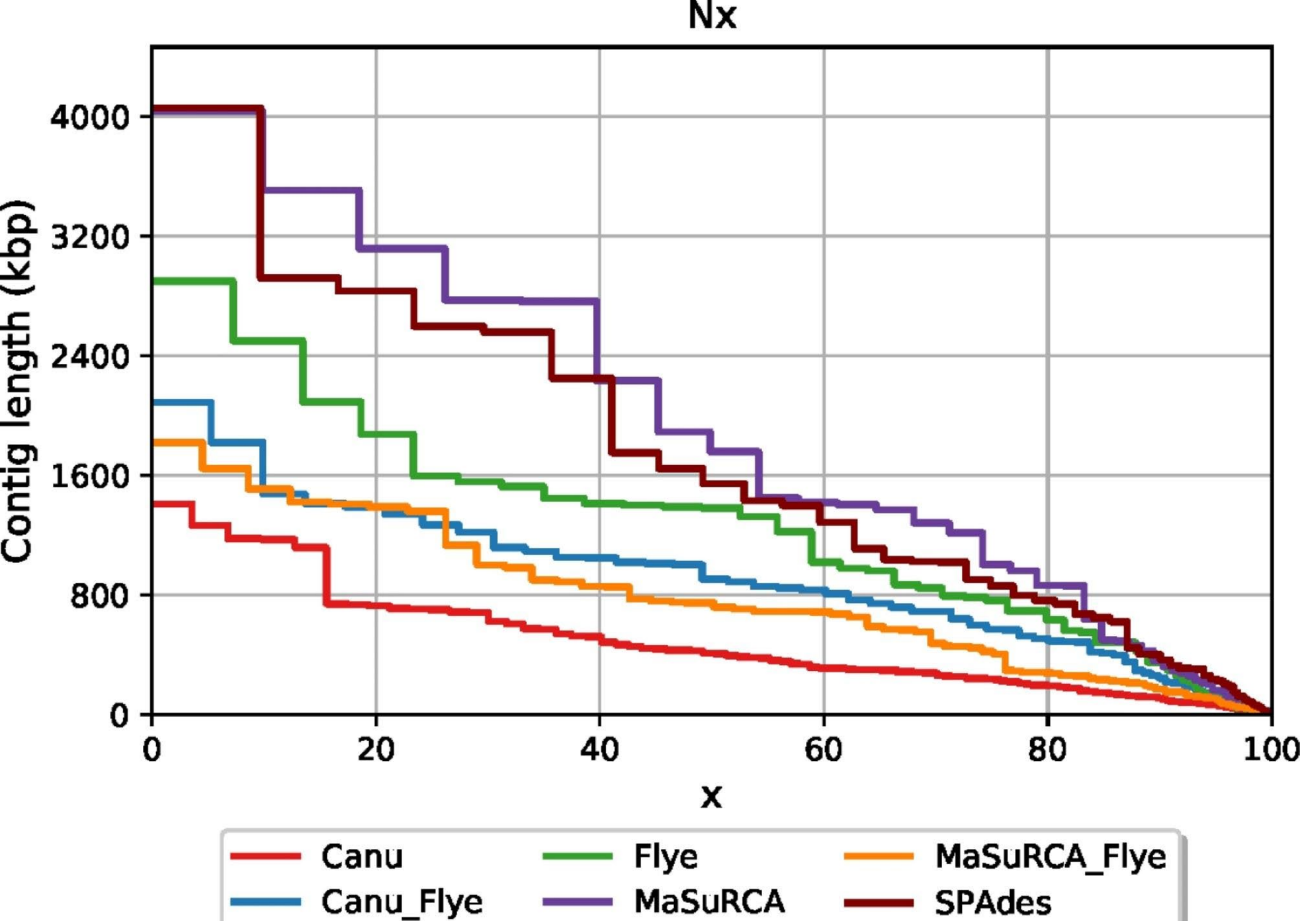
Quast Nx statistics for nuclear genome assemblies of PAR3 by different assembly platforms

Assembly analysis by Quast [22] revealed similar GC content for all assemblies, with most ranging from 48.4 to 49.1% (Table 1). The exception to this was the SPAdes assembly, which was at 47.2%. The N50 values of Flye, MaSuRCA, and SPAdes (1.4–1.8 Mb) were noticeably higher than for Canu, MaSuRCA-Flye, and Canu-Flye (0.4-9 Mb). Notably, the N50 value for Canu was just over half of the next lowest, MaSuRCA-Flye (Table 1). When comparing Nx values of all the assemblies, it was clear that MaSuRCA and SPAdes produced the most contiguous assemblies, though MaSuRCA was slightly better than SPAdes (Fig. 1). L50 values of the assemblies show that the MaSuRCA and SPAdes produced the only assemblies with possess single digit values, 8 and 9, respectively, with other assemblies possessing values of 12–29 (Table 1). The SPAdes and MaSuRCA assemblies also contained a 4 Mb scaffold, the largest single contig/scaffold reported in any of the assemblies.

**Table 1:** Quast statistics for nuclear genome assemblies. Quast statistics of polished nuclear genome assemblies by Canu, Flye, MaSuRCA and SPAdes

	Canu	Flye	Canu-Flye	MaSuRCA	MaSuRCA-Flye	SPAdes
**Size (bp)**	39,406,385	40,120,152	39,404,652	40,741,375	40,283,886	41,910,339
**Contigs**	199	114	95	121	123	99
**Scaffolds**	-	-	-	112	-	85
**Largest contig/scaffold**	1,408,620	2,898,868	2,086,106	4,033,039	1,820,786	4,058,310
**GC %**	49.05%	48.65%	49.04%	48.37%	48.53%	47.16%
**L50**	29	12	16	8	18	9
**L75**	100	22	63	54	35	70
**N50**	413,467	1,382,802	906,246	1,757,915	749,039	1,544,774
**N75**	114,775	764,355	184,637	184,782	422,163	148,461

To obtain an estimate of genome completeness, all assemblies were subject to analysis by BUSCO [23], utilizing Augustus [24] to search for BUSCOs. This analysis revealed significant disparities between the ONT-only assemblers and the hybrid assemblers, with Canu, Flye, and Canu-Flye assemblies ranging from 29 to 60% completion, and the MaSuRCA, MaSuRCA-Flye, and SPAdes ranging from 99.5 to 99.7% completeness (Table 2). The assembly of Canu-corrected ONT reads by Flye produced a higher score (60%) than Canu (50%) or Flye (29%) individually. To increase the fidelity of all assemblies, Pilon [15] was used with the Illumina reads. After polishing, the BUSCO scores of the Canu, Flye, and Canu-Flye assemblies improved dramatically, with the Canu assembly reaching a completeness of 99.4%, the Flye assembly improving to 99%, and the Canu-Flye assembly obtaining a completeness of 99.6% (Table 2). The hybrid assemblies all slightly improved to 99.7% completion after polishing with the exception of the SPAdes assembly, which was already at 99.7% prior to polishing. Collectively, all assemblies possessed more than 99% of BUSCOs after polishing, but hybrid assembly scores were consistently higher than ONT-only assemblies (Table 2). Ultimately, MaSuRCA produced the best assembly statistics and had the co-highest BUSCO completion scores and was selected for phylogenetic analysis and annotation.

**Table 2:** BUSCO analysis of nuclear genome assemblies. BUSCO analysis of initial and short-read polished nuclear genome assemblies by Canu, Flye, MaSuRCA and SPAdes

	Canu	Flye	Canu-Flye	MaSuRCA	MaSuRCA-Flye	SPAdes	
Complete BUSCOs	2244 (50%)	1286 (28.6%)	2692 (59.9%)	4475 (99.6%)	4471 (99.5%)	4479 (99.7%)	**Unpolished**
- Single-copy	2241 (49.9%)	1285 (28.5%)	2686 (59.8%)	4457 (99.2%)	4457 (992%)	4466 (99.4%)	
- Duplicated	3 (0.1%)	1 (0.1%)	6 (0.1%)	18 (0.4%)	14 (0.3%)	13 (0.3%)	
- Fragmented	822 (18.3%)	910 (20.2%)	721 (16%)	8 (0.2%)	8 (0.2%)	5 (0.1%)	
Missing BUSCOs	1428 (31.7%)	2298 (51.2%)	1081 (24.1%)	11 (0.2%)	15 (0.3%)	10 (0.2%)	
Complete BUSCOs	4467 (99.4%)	4449 (99.0%)	4479 (99.6%)	4482 (99.7%)	4479 (99.7%)	4479 (99.7%)	**Polished**
- Single-copy	4455 (99.1%)	4436 (98.7%)	4464 (99.3%)	4463 (99.3%)	4465 (99.4%)	4466 (99.4%)	
- Duplicated	12 (0.3%)	13 (0.3%)	15 (0.3%)	19 (0.4%)	14 (0.3%)	13 (0.3%)	
- Fragmented	12 (0.3%)	20 (0.4%)	4 (0.1%)	3 (0.1%)	3 (0.1%)	5 (0.1%)	
Missing BUSCOs	15 (0.3%)	25 (0.6%)	11 (0.3%)	9 (0.2%)	12 (0.2%)	10 (0.2%)	

**Fig. 2:**
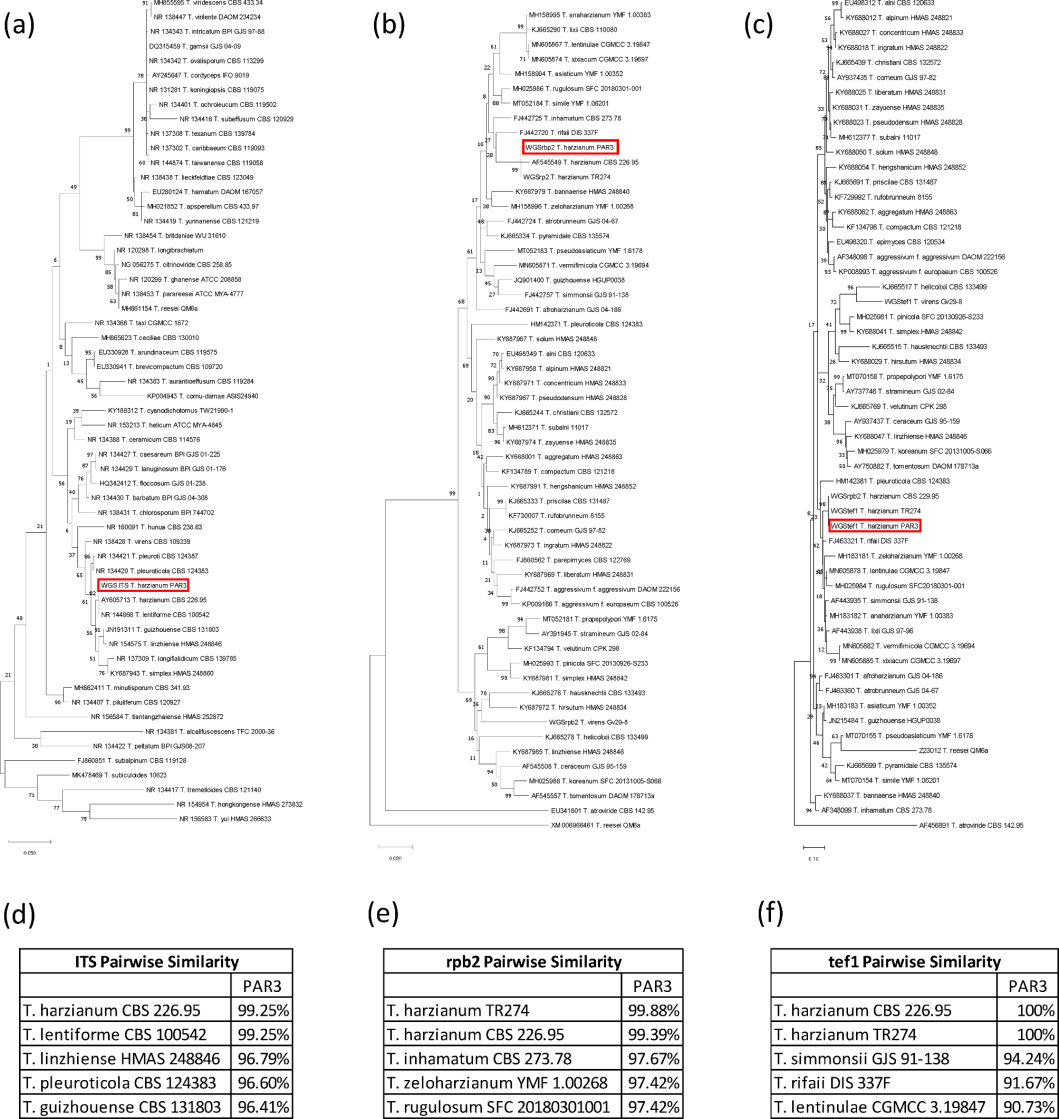
Phylogenetic trees and highest pairwise similarity matches of PAR3 barcoding sequences. The ITS (a,d), rpb2 (b,e), and tef1 (c,f) gene fragments were used for analysis. The trees were constructed in MEGA X, which used the Maximum Likelihood method and Tamura-Nei model. The percentage of trees in which the associated taxa clustered together is shown next to the branches. Initial trees for the heuristic search were obtained automatically by applying Neighbor-Join and BioNJ algorithms to a matrix of pairwise distances estimated using the Tamura-Nei model, and then selecting the topology with superior log likelihood value. The tree is drawn to scale, with branch lengths measured in the number of substitutions per site. Pairwise similarities of ITS (d), rpb2 (e), and tef1 (f) from PAR3 to other *Trichoderma* species were obtained from Clustal Omega (Maderia et al., 2022) and the five most similar species to PAR3 are shown with *Trichoderma harzianum* species bolded

Previously, it was determined that PAR3 was a strain of *Trichoderma harzianum* [5], but new recent and comprehensive guidelines were put forth by [19] for the identification of *Trichoderma* spp., which include the phylogenetic analysis of the ITS, *rpb2*, and *tef1* barcoding sequences. The MaSuRCA assembly possessed 17 copies of ITS, and all were identical except for one copy that possessed a single nucleotide mismatch. The ITS region is found between coding regions of rRNA subunits, and further investigation revealed that there were 17 complete rRNAs in PAR3. Fragments of the ITS, *rpb2*, and *tef1* regions of PAR3 were aligned to those of other *Trichoderma* species from the *Harzianum* clade, and phylogenetic trees and pairwise similarities of these alignments were calculated. All three of the barcoding sequences from PAR3 shared the greatest identity with two other confirmed strains of *T. harzianum*, with the PAR3 ITS sharing over 99% identity, *rpb2* sharing over 99% similarity, and *tef1* possessing 100% identity to the respective barcodes from *T. harzianum* CBS 226.95 and *T. harzianum* TR274 (Fig. 2). These results satisfy the requirements laid out by Cai and Druzhinina [19] for identification of PAR3 as *T. harzianum*.

**Fig. 3:**
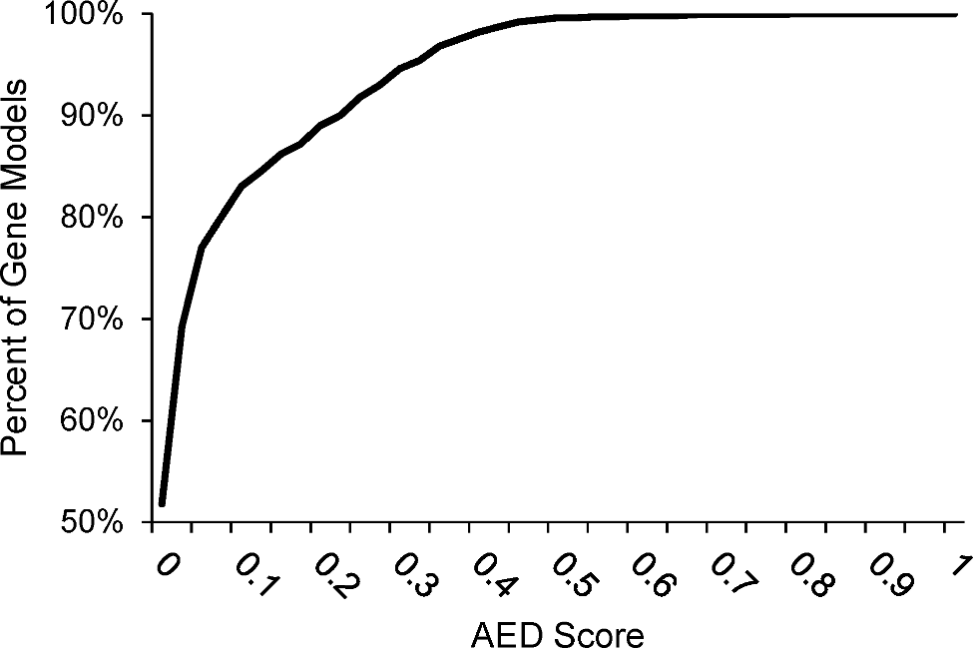
AED score of gene models produced by Maker. X-axis shows AED score, and Y-axis shows the percent of genes that are lesser or equal to AED scores

Having confirmed the identify of *T. harzianum* PAR3, Maker3 [16] was used to generate gene models with publicly available mRNA and protein data of RefSeq *Trichoderma* spp. and *ab-initio* gene prediction by SNAP [25] and Augustus. This yielded 12,074 protein-encoding gene models with an average length of 1820 nucleotides and an average of 3.1 exons per gene (Table 3). Of these gene models, 99.5% possessed an AED score of less than 0.5, 91.8% were less than 0.25, and 82.8% had an AED score of less than 0.1 (Fig. 3). Analysis by tRNAScan-SE [26] revealed the PAR3 nuclear genome contains 210 tRNAs, with four of these likely to be non-functional. The number of models found in PAR3 was less than the 14,064 reported for *T. harzianum* strain CBS 226.95 and the 13,925 gene models from *T. harzianum* TR274, but was within the range of genes reported for other *Trichoderma* spp. Additionally, were 5 more tRNAs found in *T. harzianum* PAR3 than in *T. harzianum* CBS 226.95.

**Table 3:** Summary statistics of *Trichoderma harzianum* PAR3 annotations

Number of genes	12,057
mean gene length	1,820
mean exons per gene	3.1
mean exon length	526
Number of tRNAs	210
Number of rRNAs	17

Functional annotation of Maker gene models by InterProScan and showed that 8,660 protein-encoding gene models from *T. harzianum* PAR3 possessed at least one InterPro domain. Similar analysis of publicly available data from *T. harzianum* CBS 226.95 showed 8,896 gene models that contained at least one functional InterPro domain. Comparison of classification and number of protein domains between the CBS 226.95 and PAR3 strains few instances of substantial differences (Table 4). In total, the *T. harzianum* CBS 226.95 genome possessed a little over 100 more identified domains than the PAR3 genome, but there were few differences in the number of identified protein domains. Notably, there were 9 more “Protein kinase” (IPR000719), 16 more “ABC transporter-like, ATP-binding” (IPR003439), and 23 more “NACHT nucleoside triphosphatase” (IPR007111) domains present in the CBS 226.95 strain than the PAR3 strain. When comparing genes that belong to InterPro gene families, even fewer differences were found, *T. hazianum* CBS 226.95 genome containing 3,611 gene models that correspond to known InterPro families and the *T. hazianum* PAR3 genome containing 3,576 (Table 5). Few substantial differences between these *T. hazianum* strains were found at the gene family level, with the biggest differences being that the CBS 226.95 strain contains 5 more “Fungal transcription factor” (IPR021858) genes and 4 more “Oligopeptide transporter” genes than the PAR3 strain.

**Table 4:** Top InterPro domains in *T. harzianum* PAR3 and *T. harzianum* CBS 226.95

InterPro ID	Domain Description	PAR3	CBS 226.95
IPR001138	Zn(2)-C6 fungal-type DNA-binding	365	383
IPR007219	Transcription factor domain, fungi	255	251
IPR020683	Domain of unknown function DUF3447	124	121
IPR000719	Protein kinase domain	103	112
IPR000073	Alpha/beta hydrolase fold-1	94	94
IPR010730	Heterokaryon incompatibility	88	92
IPR013154	Alcohol dehydrogenase-like, N-terminal	91	90
IPR013149	Alcohol dehydrogenase-like, C-terminal	87	88
IPR008030	NmrA-like domain	79	78
IPR003439	ABC transporter-like, ATP-binding	61	77
IPR001650	Helicase, C-terminal	74	74
IPR007111	NACHT nucleoside triphosphatase	47	70
IPR000182	GNAT domain	63	66
IPR002938	FAD-binding domain	64	62
IPR000504	RNA recognition motif	59	58
	*Total number of identified domains*	*8868*	*8983*

**Table 5:** Top InterPro gene families in *T. harzianum* PAR3 and *T. harzianum* CBS 226.95

InterPro ID	Family Description	PAR3	CBS 226.95
IPR011701	Major facilitator	265	266
IPR002347	Short-chain dehydrogenase/reductase SDR	155	156
IPR001128	Cytochrome P450	115	117
IPR021858	Fungal transcription factor	110	115
IPR005828	Major facilitator, sugar transporter-like	96	96
IPR003663	Sugar/inositol transporter	68	69
IPR021765	Mycotoxin biosynthesis protein UstYa-like	38	37
IPR001806	Small GTPase	26	26
IPR001757	P-type ATPase	22	22
IPR001753	Enoyl-CoA hydratase/isomerase	20	21
IPR020946	Flavin monooxygenase-like	21	21
IPR002293	Amino acid/polyamine transporter I	20	20
IPR021833	Protein of unknown function DUF3425	17	19
IPR004813	Oligopeptide transporter	13	17
IPR007568	RTA-like protein	17	17
	*Total number of genes belong to any family*	*3576*	*3611*

Since secondary metabolites are a major source of anti-microbial natural products, we used antiSMASH [27] to find putative polyketide synthases (PKS), non-ribosomal peptide synthases (NRPS), or hybrid polyketide synthase non-ribosomal peptide synthases (PKS-NRPS) within *T. hazianum* PAR3. Collectively, these genes produce a wide array of small peptides and molecules that are often involved in the synthesis of metabolites that have anti-microbial activity. There were 10 complete NRPS within the *T. hazianum* PAR3 genome, as well as 9 additional NRPS-like fragments. A total of 20 type I PKS and 9 terpene synthase genes were also found within the genome. Lastly, 6 different PKS-NRPS hybrid genes were found within the genome, including a 12 NRPS-module-containing gene and an 18 NRPS-module containing gene. Two additional multi-gene clusters were predicted to form modular PKS-NRPS. The only difference between the number of these genes between *T. hazianum* PAR3 and *T. hazianum* CBS 226.95 was that the latter possesses one less NRPS cluster, however upon closer inspection, one of the NRPS clusters in *T. hazianum* CBS 226.95 appears to have been annotated as two separate clusters in *T. hazianum* PAR3.

To attempt to gain a better understanding of the differences between *T. harzianum* PAR3 and other *T. harzianum* strains, compared predicted proteins among the PAR3, CBS226.95, and TR274 strains. We found the majority of the 12,074 gene models in PAR3 were homologous to at least one gene in the other two strains (Table 6). However, there were a small number of genes in the PAR3 strain, 845 and 1,256, that did not share homology with genes in the CBS226.95 or TR274 strains, respectively. Overall, there were 221 genes in PAR3 that are not homologous to either any gene in either strain, and appear unique to PAR3. Out of these, 125 contained known InterPro domains, with the most abundant types of domains displayed in Table 7.

**Table 6:** Comparison of *T. harzianum* PAR3 genes to other *T. harzianum* strains

	CBS 226.95	TR274
**Equivalent ** **genes in PAR3**	11,229 (93.0%)	10,818 (89.6%)
**PAR3 genes ** **absent from strain**	845 (7%)	1256 (10.4%)
**PAR3 genes absent ** **from both strains**	221 (1.8%)	

Table 6 The number of genes in *T. harzianum* PAR3 that are present or absent in other *T. harzianum* strains, with percentage of total genes in the PAR3 strain shown underneath.

**Table 7:** Most abundant InterPro domains of unique PAR3 genes

InterPro ID	Type	Domain/Family Description	Gene #
IPR001138	Domain	Zn(2)-C6 fungal-type DNA-binding domain	5
IPR020683	Domain	Domain of unknown function DUF3447	5
IPR000073	Domain	Alpha/beta hydrolase fold-1	4
IPR008030	Domain	NmrA-like domain	3
IPR013149	Domain	Alcohol dehydrogenase-like, C-terminal	3
IPR013154	Domain	Alcohol dehydrogenase-like, N-terminal	3
IPR023631	Domain	Amidase signature domain	3

## Discussion

Four different popular assembly platforms, Canu, Flye, MaSuRCA, and SPAdes, and two of their combinations, Canu-Flye and MaSuRCA-Flye, were tested to see which produced the best draft nuclear genome assembly of *T. harzianum* PAR3. Of these, SPAdes and MaSuRCA produced the best initial assembly statistics (Fig. 1; Table 1), which was expected as these platforms used both long and short reads for assembly. Initial BUSCO scores were poor for ONT-only assemblies, while scores for hybrid assemblers were all greater than 99% (Table 2). This was not surprising, as ONT reads are known to be lower fidelity than Illumina reads and could have made identification of BUSCOs more difficult. In this case, the relatively low coverage (28x) of ONT reads likely exacerbated this issue. The use of higher coverage ONT datasets may improve BUSCO scores of ONT-only assemblies, though the high error rate of ONT sequencing would likely require substantially greater coverage to compete with hybrid assemblies utilizing high-fidelity Illumina reads. Flye assembly of error-corrected ONT reads from Canu, as opposed to raw ONT reads, resulted in more than double the number of complete BUSCOs (Table 2), emphasizes the importance of read fidelity on genome assembly. After Polishing with Pilon, the ONT-only assemblies displayed dramatic increases in BUSCO scores that were comparable to that of the hybrid assemblers (Table 2; Fig. 2). However, taking both the assembly statistics and BUSCO scores into account, the SPAdes and MaSuRCA assemblies deemed to be of the highest quality. Despite similar qualities, the assembly statistics were slightly better for the MaSuRCA assembly, and thus this was chosen as the assembly to be used for phylogenetic analysis and annotation.

When pulling out the ITS barcoding sequences for phylogenetic analysis of PAR3, the MaSuRCA assembly was found to possess 17 complete and 2 partial ITS sequences. Accordingly, 17 complete rRNA genes were found, with 2 partial rRNA genes lacking the 18 S sequence and part of the ITS region. This was not surprising, as it is known that copy number of rRNA/ITS in fungi can range anywhere from approximately 14 − 1,442 within fungal genomes [28]. However, none of the other assemblies produced more than one ITS, which suggests that the structural composition of the MaSuRCA assembly was superior to that of the others. This may be due to the strategy of the MaSuRCA assembler, which creates large “mega-reads” based on the combination of long reads and “super-reads” created from short reads [14].

Phylogenetic analysis of the MaSuRCA PAR3 assembly found that the ITS, *rpb2*, and *tef1* sequences of PAR3 were highly homologous to those of identified strains of *T. harzianum* (Fig. 2). Cai and Druzhinina [19] outlined distinct criteria for identification of Trichoderma *spp*., which is especially difficult given the close identity of Trichoderma belonging to the *Harzianum* clade [18]. As per Cai and Druzhinina [19], more than 76% identity of an isolate’s ITS sequence to other *Trichoderma* spp. is required to confirm it belongs to the *Trichoderma* genus, and more than 99% and 97% identity of *rpb2* and *tef1* sequences, respectively, is required to identify an isolate at the species level. All three sequences extracted from PAR3 were more than 99% identical to those of known strains of *T. harzianum*, satisfying the requirements to confirm PAR3 as *Trichoderma harzianum*.

Annotation of the PAR3 draft genome resulted in good AED values of predicted genes (Fig. 3), though about 2,000 less protein-encoding genes were found in PAR3 compared to other confirmed *T. harzianum* strains, CBS226.95 and TR274 (Table 3). Despite this difference, there were relatively few differences in the overall number and type of InterPro domains or gene family members was found between the type strain of *T. harzianum*, CBS 226.95, and *T. harzianum* PAR3. Even fewer differences in the number or modular structure of PKS, NRPS, and terpene synthase gene clusters between these strains were found. Additionally, 5 more tRNAs were found in the *T. harzianum* PAR3 genome compared to *T. harzianum* CBS 226.95, though two of the identified tRNAs are likely to be non-functional. *T. harzianum* CBS 226.95 and TR274 strains were isolated from Europe and South America, respectively, and previous comparison of these strains revealed 1,699 (12%) and 1,419 (10.1%) genes were unique to each strain, respectively [20]. Similarly, this analysis revealed that the *T. harzianum* PAR3, isolated in North America, has 845 (7%) and 1,256 (10.4%) genes that are absent in the CBS 226.95 and TR274 strains, respectively. Out of these, there were 221 (1.8%) genes in the PAR3 strain that were not present in either of the other strains, with 125 genes possessing predicted InterPro protein domains. These PAR3-unique genes consisted of 94 different InterPro domains, with multiple genes possessing the most overall abundant domains in the PAR3 strain (Tables 4 and 7).

## Conclusion

Several popular genome assemblers were tested for their ability to perform genome assemblies of *T. harzianum* PAR3 with 28x ONT and 518x Illumina reads. The hybrid assemblers SPAdes and MaSuRCA produced the best assembly statistics and were the most complete. Of these, the MaSuRCA assembly was determined to be the highest quality assembly of those obtained. Subsequent annotation of this 40.7 Mb genome assembly produced 12,057 gene models and 210 tRNAs, and putative function of these genes was assigned. PAR3 putatively possesses the ability to inhibit growth of several fungal canker pathogens of grapevine, and thus represents a potentially useful resource for grapevine growers [5]. In addition to the previous reporting of the mitochondrial genome of PAR3 [5], this draft nuclear genome assembly and its annotation will aid investigations into its ability to act as a potential biocontrol agent of grapevine and into its synthesis of anti-microbial metabolites.

## Methods

### Fungal material

The PAR3 strain of *T. harzianum* was isolated from the roots of a Scarlet Royal grapevine grafted to Freedom rootstock in a vineyard in Parlier, CA, USA. Cultures of PAR3 were grown in potato dextrose broth (Difco Laboratories, Detroit, MI, U.S.A.) placed on a shaker at 150 rpm under ambient light at 26 °C for one week prior to DNA extraction.

### DNA extraction and sequencing

Genomic DNA was extracted from a PAR3 liquid culture with the Plant Mini Kit from Marchery-Nagel (Bethlehem, PA, U.S.A.), according to manufacturers’ recommendations. A Qubit fluorometer and a Qubit 1X dsDNA HS Assay Kit, from Invitrogen (Carlsbad, CA, U.S.A.), were used to determine DNA quantity, and then genomic DNA was amplified by a Illustra GenomiPhi version 2 amplification kit (GE Healthcare, Waukesha, WI, U.S.A.). For short-read sequencing, amplified genomic DNA was used to construct a 150 paired-end library using a HiSeq PE150 kit (Illumina, San Diego, CA, U.S.A.), and then sequenced on an Illumina HiSeq 2500 with 2 × 150 bp paired-end format. For long-read sequencing, amplified genomic DNA was used to construct a sequencing library using a 1D Native barcoding genomic DNA kit from Oxford Nanopore Technologies (ONT) (Alameda, CA, U.S.A.), and then this library was sequenced by an ONT minION system. Illumina sequencing produced 141,612,983 paired-end reads for a total of 104.68 Gb (Q > 30) and 518X predicted coverage. ONT sequencing produced 126,156 reads ranging from 60 to 65,000 bp in length, with an average length of 9,020 bp (N50 = 7,640 bp), for a total of 1.06 Gb (Q > 20) and 28X predicted coverage.

### Genome assembly and evaluation

Long read-based assembly of ONT-produced data was performed by both Canu (v2.2) [10] and Flye (v2.8.3) [11]. Default parameters were used for both Flye and Canu assemblies, except the estimated genome size, which was set to 41 Mb for both. Canu utilized raw fastq reads, whereas Flye utilized corrected reads obtained from Canu. Hybrid assembly utilizing reads obtained from both Illumina and ONT sequencing was performed utilizing both SPAdes (v3.14.0) [29] and MaSuRCA (v4.0.1) [14]. SPAdes and MaSuRCA assemblies were performed with fastq Illumina and ONT reads, and largely utilized default settings. Non-default settings included using the “isolate” option in SPAdes and K-mer sizes of 21, 33, 55, 77, 99, and 127. JF hash size in MaSuRCA was set to 8 × 10^8^. All assemblies were subject to polishing with Pilon (v1.23) [15] utilizing Illumina reads aligned to the respective assemblies utilizing Bowtie2 [30] with the “very careful” option selected. All genome assemblies were subject to analysis via Quast (v5.0.2) [21] and BUSCO (v5.2.2) [23] to determine assembly statistics and their completeness. BUSCO analysis utilized Augustus [24], using “*Fusarium”* parameters, to search for BUSCOs from the “hypocreales_odb10” database.

### Phylogenetic analysis

ITS, *rpb2*, and *tef1* sequences from PAR3 were identified in the MaSuRCA assembly by using BLAST+ (v2.11.0) [31] to search against the known ITS (AF510497.1), *rpb2* (XM_006966461.1), and *tef1* (XM_006963994.1) sequences of *Trichoderma reseii* strain QM6a available on NCBI. Additional publicly available sequences from other *Trichoderma* spp. were also obtained from established “type” strains on NCBI. The ITS56 data set provided by Cai and Druzhinina [19] was utilized for phylogenetic analysis of the ITS of PAR3. All *tef1* sequences included in phylogenetic analyses were trimmed using the online *Tricho*Mark 2020 tool [32], with the 4th intron used for phylogenetic analysis. *rpb2* sequences were manually trimmed according to Cai and Druzhinina [19]. Pairwise similarity scores were obtained by Clustal Omega [33]. Phylogenetic trees were constructed by MEGA X [34] using the Maximum Likelihood method and Tamura-Nei model [35].

### Genome annotation and analysis

The MaSuRCA assembly was selected for genome annotation by Maker (v3.01.03) [16]. RepeatModeler (v2.0.2) [36] was used to generate a library repeat library prior to analysis by Maker. RepeatMasker (v4.0.1) [37] was used within Maker to mask low complexity and repeat regions of the assembly. Initial Maker gene predictions were made by utilizing publicly available RNA transcripts and proteins from the RefSeq assembly of *T. harzianum* CBS 226.95 (GCF_003025095.1), as well as proteins from other RefSeq *Trichoderma* assemblies, including, *T. virens* (GCF_000170995.1), *T. gamsii* (GCF_001481775.2), *T. asperellum* (GCF_003025105.1), *T. atroviride* (GCF_000171015.1), *T. cintrinoviride* (GCF_003025115.1), and *T. reesei* (GCF_000167675.1). SNAP (v2013.11.29) [25] and Augustus (v3.4.0) [24] gene predictors were utilized within Maker to inform gene models. SNAP and Augustus were trained and optimized after each round of Maker (2 rounds of *ab-initio* training, 3 rounds of Maker total). Augustus was trained via BUSCO (v5.2.2) [23]. Gene models produced by Maker were then functionally annotated by InterProScan (v5.56) [17]. Identification of tRNAs was determined by tRNAScan-SE (v2.0.5) [26]. Detection of PKS, NRPS, and terpene synthase genes was performed with antiSMASH (6.1.1) [27] using the MaSuRCA assembly and Maker3 annotations. For comparison, this same analysis was also performed for *T. harzianum* CBS 226.95 (GCF_003025095.1) using genome sequences and annotation features. Proteins from the publicly available *T. harzianum* CBS 226.95 (GCF_003025095.1) and *T. harzianum* TR274 (GCA_002838845.1) accessions were functionally annotated as described above, and equivalent genes between these strains and PAR3 were identified using BLAST+ (v2.11.0) [31], with proteins matches greater than 95% identity and e-values less than 0.00001 considered equivalent.

## Data Availability

The *Trichoderma harzianum* PAR3 nuclear genome sequence has been submitted at DDBJ/EMBl/GenBank under the project number PRJNA880851.
